# SARS-CoV-2 infection and vaccination status in six ethnic groups in Amsterdam, The Netherlands, May to November 2022

**DOI:** 10.1017/S0950268825000056

**Published:** 2025-01-23

**Authors:** Sophie L. Campman, Anders Boyd, Janke Schinkel, Liza Coyer, Charles Agyemang, Henrike Galenkamp, Anitra D.M. Koopman, Felix P. Chilunga, Jelle Koopsen, Aeilko H. Zwinderman, Suzanne Jurriaans, Karien Stronks, Maria Prins

**Affiliations:** 1 Department of Infectious Diseases, Public Health Service of Amsterdam, Amsterdam, The Netherlands; 2 Amsterdam UMC location University of Amsterdam, Infectious Diseases, Amsterdam, The Netherlands; 3 Amsterdam Institute for Immunology and Infectious Diseases, Amsterdam, The Netherlands; 4 Stichting HIV Monitoring, Amsterdam, The Netherlands; 5 Amsterdam UMC location AMC, University of Amsterdam, Department of Medical Microbiology and Infection Prevention, Amsterdam, The Netherlands; 6 Department of Public and Occupational Health, Amsterdam Public Health Research Institute, Amsterdam UMC, University of Amsterdam, Amsterdam, The Netherlands; 7 Department of Medicine, Division of Endocrinology, Diabetes and Metabolism, Johns Hopkins University School of Medicine, Baltimore, MA, USA; 8 Amsterdam Public Health, Health Behaviors and Chronic Diseases, Amsterdam, The Netherlands; 9 Amsterdam UMC location University of Amsterdam, Clinical Epidemiology, Biostatistics and Bioinformatics, Amsterdam, The Netherlands

**Keywords:** antibodies, ethnicity, immunity, SARS-CoV-2, seroprevalence, vaccination

## Abstract

We studied severe acute respiratory syndrome coronavirus 2 (SARS-CoV-2) infection and vaccination status among six ethnic groups in Amsterdam, the Netherlands. We analysed participants of the Healthy Life in an Urban Setting cohort who were tested for SARS-CoV-2 spike protein antibodies between 17 May and 21 November 2022. We categorized participants with antibodies as only infected, only vaccinated (≥1 dose), or both infected and vaccinated, based on self-reported prior infection and vaccination status and previous seroprevalence data. We compared infection and vaccination status between ethnic groups using multivariable, multinomial logistic regression. Of the 1,482 included participants, 98.5% had SARS-CoV-2 antibodies (*P* between ethnic groups = 0.899). Being previously infected and vaccinated ranged from 41.5% (95% confidence interval (CI) = 35.0–47.9%) in the African Surinamese to 67.1% (95% CI = 59.1–75.0%) in the Turkish group. Compared to participants of Dutch origin, participants of South-Asian Surinamese (adjusted odds ratio (aOR) = 3.31, 95% CI = 1.50–7.31)), African Surinamese (aOR = 10.41, 95% CI = 5.17–20.94), Turkish (aOR = 3.74, 95% CI = 1.52–9.20), or Moroccan (aOR = 15.24, 95% CI = 6.70–34.65) origin were more likely to be only infected than infected and vaccinated, after adjusting for age, sex, household size, trust in the government’s response to the pandemic, and month of study visit. SARS-CoV-2 infection and vaccination status varied across ethnic groups, particularly regarding non-vaccination. As hybrid immunity is most protective against coronavirus disease 2019, future vaccination campaigns should encourage vaccination uptake in specific demographic groups with only infection.

## Introduction

Early in the coronavirus disease 2019 (COVID-19) pandemic, it became apparent that ethnic minority populations were at increased risk of infection with severe acute respiratory syndrome coronavirus 2 (SARS-CoV-2) and severe progression of COVID-19, including hospitalization and mortality [1]. The risk of SARS-CoV-2 infection and severe disease progression can be effectively reduced by immunity acquired through infection, vaccination, or both [2, 3].

In Amsterdam, the Netherlands, data from the multi-ethnic Healthy Life in an Urban Setting (HELIUS) cohort identified ethnic differences in SARS-CoV-2 infections in the pre-vaccination era. Between June and October 2020, following the first wave of the Dutch epidemic, individuals of Ghanaian ethnic origin had a higher seroprevalence than individuals of Dutch, Surinamese (South-Asian and African), Turkish, or Moroccan origin [4]. Between November 2020 and March 2021 (i.e., the second wave), differences in incidence became wider for all other ethnic minority groups compared to the Dutch origin group. The estimated cumulative incidence of infection remained the highest in individuals of Ghanaian origin (64.4%), compared to 15.9% in the group of Dutch origin [5]. When the primary SARS-CoV-2 vaccination series became available in early 2021, data from this cohort showed that the uptake of at least one dose was lower in most ethnic minority groups compared to individuals of Dutch origin by mid-2021 [6].

By mid-2022, much of the Dutch population had been infected with SARS-CoV-2, partly due to the highly transmissible Omicron variant [7], and the abolishment of most mitigation measures, such as social distancing [8]. Moreover, the entire Dutch population had the opportunity to receive both primary and booster vaccinations. Previous studies have demonstrated that hybrid immunity, which is a combination of antibodies acquired through prior SARS-CoV-2 infection and vaccination, provides greater and more durable protection against severe COVID-19 than natural or vaccine-induced immunity alone, underscoring the importance of vaccination uptake even after a previous infection [9, 10]. However, it is unknown whether the distribution of protection through hybrid immunity, prior infection, or vaccination alone differs between ethnic groups. Understanding these potential ethnic differences is crucial in identifying potential inequalities in protection against severe COVID-19 outcomes. This knowledge can guide targeted public health interventions to ensure equitable protection and address future health inequities.

This study aimed to describe the prevalence of anti-spike SARS-CoV-2 antibodies among people of Dutch, South-Asian Surinamese, African Surinamese, Ghanaian, Turkish, and Moroccan origin in Amsterdam, the Netherlands, and to compare the SARS-CoV-2 infection and vaccination status (i.e., only prior infection, only vaccination, or both infection and vaccination) among people with SARS-CoV-2 antibodies between ethnic groups.

## Methods

### Study design and population

We used data from the HELIUS study, which is a population-based multi-ethnic prospective cohort study conducted in Amsterdam that focuses on the causes of potential ethnic disparities in cardiovascular disease, mental health, and infectious diseases. Detailed procedures have been previously described [11]. Briefly, the parent HELIUS cohort comprises 24,780 adult individuals of Dutch, Surinamese, Ghanaian, Turkish, and Moroccan origin living in Amsterdam who were included between January 2011 and December 2015. Individuals were randomly sampled, stratified by ethnic origin, through the municipality register of Amsterdam, and invited to participate [11, 12]. This register contains data on country of birth of citizens and their parents, which we used to determine ethnic origin. Country of birth is a widely accepted and stable indicator for ethnic origin in the Netherlands, while Dutch studies have shown high correlation between country of birth and self-identified ethnicity among Turkish, Moroccan, and Surinamese groups [12]. We defined ethnic origin groups other than Dutch as: (1) the individual, and at least one parent, was not born in the Netherlands (first-generation migrants), and (2) the individual was born in the Netherlands, but both parents were not (migrants’ offspring). Given the ethnic heterogeneity of the Surinamese population [11, 12], we further classified participants with a Surinamese background into African, South-Asian, Javanese, or ‘other’ based on self-report during the baseline questionnaire. Participants completed a questionnaire and underwent physical examination during which biological samples were obtained. The HELIUS study was approved by the Academic Medical Center Ethical Review Board, and written informed consent was obtained from all participants [11].

Shortly after the start of the COVID-19 pandemic, participants of the parent HELIUS cohort who were still in follow-up and of Dutch, South-Asian Surinamese, African Surinamese, Ghanaian, Turkish, or Moroccan origin were randomly selected within each ethnic group and were asked to participate in a three-visit longitudinal COVID-19 substudy [4]. The first COVID-19 substudy visit took place between 24 June and 9 October 2020. Participants of the first visit were invited to participate in the second visit between 23 November 2020 and 4 June 2021 and the third visit between 17 May and 21 November 2022. This study included participants of the third COVID-19 substudy visit. During all three visits, blood samples were obtained via venipuncture, stored at −20°C, and were tested for SARS-CoV-2-specific antibodies. Trained interviewers also administered questionnaires on items, such as SARS-CoV-2 exposure, testing, infection history, perceptions, and vaccination uptake. During the third substudy visit, participants who indicated that they could not visit the study site due to long COVID were visited at home to limit selection bias due to post-COVID-19 complications.

### Study outcomes

First, we described the SARS-CoV-2 antibody test result (positive versus negative) during the third COVID-19 substudy visit. SARS-CoV-2-specific antibodies were determined using the WANTAI SARS-CoV-2 Ab enzyme-linked immunosorbent assay (ELISA) (Wantai Biological Pharmacy Enterprise Co., Beijing, China). This ELISA detects immunoglobulin A (IgA), IgM, and IgG against the receptor-binding domain of the spike protein of SARS-CoV-2 [13]. Even though this test cannot discriminate between antibodies acquired through infection versus vaccination, the sensitivity of the WANTAI ELISA is higher compared to other assays for detection of SARS-CoV-2 antibodies [14].

Second, we defined SARS-CoV-2 infection and vaccination status as being (i) only vaccinated, (ii) only previously infected, or (iii) both infected and vaccinated, among those who tested positive for SARS-CoV-2 antibodies during the third COVID-19 substudy visit. Vaccination status was defined as receiving at least one vaccine dose based on self-report during the third visit. For unvaccinated participants, prior infection was based on a positive antibody test at the third visit. For vaccinated participants, prior infection was based on a positive antibody test from the second (November 2020 to June 2021) or, if unavailable, the first visit (June to October 2020). Nearly all HELIUS participants had their second visit before April 2021, when vaccines were only available to healthcare workers and individuals aged >75 years [15]. During this period, most participants were ineligible for vaccination. We then excluded the few participants who reported receiving vaccination before this visit. When previous antibody test results were negative or missing, prior infection was determined by self-report at the third visit, including both confirmed (i.e., through rapid antigen test or Nucleic Acid Amplification Test by a health professional or rapid antigen self-test) and suspected (i.e., not confirmed by any test) infections. More detailed information on the classification is provided in Supplementary Method 1 and Supplementary Figure S1.

### Covariates

We previously explored a wide range of sociodemographic, psychological, and cultural determinants of SARS-CoV-2 exposure, vaccination intent, and uptake across ethnic groups [4–6, 16]. For this analysis, we selected a priori several key sociodemographic (i.e., age, sex, household size), access to healthcare (i.e., health literacy) and cultural factors (i.e., cultural orientation) based on their relevance in previous findings. We additionally included governmental trust as a structural factor driving SARS-CoV-2 vaccine hesitancy [17, 18].

We used the following data from the baseline visit of the parent HELIUS study: age (based on the municipal registry; recalculated for the third COVID-19 substudy visit), sex, number of household members, health literacy, and cultural orientation (no integration (including separation and marginalization) versus integration (also including assimilation)). More detailed information on the instruments used has been previously described [6].

From the third COVID-19 substudy visit, we used the participants’ level of trust in the response of the Dutch government in containing the SARS-CoV-2 pandemic, which was measured on a 5-point Likert scale, ranging from 1 (‘no trust at all’) to 5 (‘a lot of trust’). We categorized the scores for governmental trust into no trust (scores 1–2), neutral (3), and trust (4–5) .

### Statistical analysis

The qualitative SARS-CoV-2 antibody test results from the third COVID-19 substudy visit were described and compared between ethnic groups using Pearson’s χ^2^ test.

Among participants with antibodies, we compared the SARS-CoV-2 infection and vaccination status between ethnic groups using multinomial logistic regression. We calculated the univariable odds ratio (OR) and 95% confidence interval (CI) comparing the odds of being (1) only previously infected or (2) only previously vaccinated versus being both previously infected and vaccinated across ethnic groups. We then selected a priori several determinants of infection and vaccination status as covariates in a first model (i.e., age, sex, household size, trust in the government’s response to the pandemic) (model 1). In a second model (model 2), we included age, sex, household size, and trust in the government’s response to the pandemic, along with health literacy and cultural orientation, while excluding individuals of Dutch origin, as the available health literacy and cultural orientation data often do not apply to this group. Observations with missing values on covariates were removed from analysis. We adjusted both models for the month of study visit, as those who participated later in time had a progressively higher risk of infection or vaccination. We performed an E-value analysis to assess the minimum strength of association that a potential unmeasured confounder would need to have with both ethnicity and SARS-CoV-2 infection and vaccination status to fully explain away the observed effect [19]. We conducted a sensitivity analysis only including individuals with a SARS-CoV-2 antibody test result at all three substudy visits (Supplementary Method 2).

The SARS-CoV-2 infection and vaccination status (percentage only vaccinated, only previously infected, or both) and regression analyses accounted for sampling and were rendered representative of the population structure of Amsterdam by assigning post-stratification weights corresponding to the distribution of age and sex in the specific ethnic groups in Amsterdam (Supplementary Method 3) [4]. A *P* value <0.05 was considered statistically significant. All analyses were performed using STATA version 17.0 (College Station, TX, USA).

## Results

### Description of the study population

In total, 1,482 individuals who participated in the third substudy visit between May and November 2022 were included in analyses. Inclusion and exclusion criteria are described in Supplementary Figure S2. Detailed information on differences between participants of the parent HELIUS cohort who were included versus not included in the third COVID-19 substudy visit is presented in Supplementary Table S1. Briefly, participants included in the third visit were more likely to be of Dutch or South-Asian Surinamese origin, slightly older, more highly educated, more integrated in the host society, more likely to have adequate health literacy level, and more proficient in the Dutch language compared with those not included.

Participant characteristics are presented in Table 1. The median age was 58 years (interquartile range (IQR) 48–65), ranging between 26 and 81 years at time of participation in the third substudy visit. The majority of participants was female (57.2%). The proportion of participants with a higher educational level ranged from 10.3% in the Ghanaian group to 67.1% in the Dutch group. Compared to participants of Dutch ethnic origin, those of other than Dutch origin were more likely to live in larger households. Participants of Ghanaian origin were the most likely to trust the response of the Dutch government in containing the pandemic (78.4%), while those of Turkish origin were the least likely to have trust (33.1%).

**Table 1.** Characteristics of the HELIUS participants included in the third COVID-19 substudy visit, per ethnic group, Amsterdam, the Netherlands, 17 May 2022 to 21 November 2022

Characteristic	Total (*n* = 1,482)	Dutch (*n* = 375)	South-Asian Surinamese (*n* = 328)	African Surinamese(*n* = 279)	Ghanaian(*n* = 134)	Turkish(*n* = 181)	Moroccan(*n* = 185)	*P* value
	*n* (%)	*n* (%)	*n* (%)	*n* (%)	*n* (%)	*n* (%)	*n* (%)	
Age in years,^a^ ^,^^b^ median (IQR)	58.0 (48.0–65.0)	61.0 (51.0–69.0)	58.0 (51.0–65.0)	61.0 (51.0–67.0)	58.0 (51.0–63.0)	53.0 (44.0–60.0)	52.0 (44.0–58.0)	<0.001
<45	264 (17.8)	63 (16.8)	53 (16.2)	27 (9.7)	20 (14.9)	47 (26.0)	54 (29.2)	
45–54	316 (21.3)	52 (13.9)	71 (21.6)	57 (20.4)	28 (20.9)	50 (27.6)	58 (31.4)	
55–59	245 (16.5)	55 (14.7)	51 (15.5)	34 (12.2)	29 (21.6)	38 (21.0)	38 (20.5)	
≥60	657 (44.3)	205 (54.7)	153 (46.6)	161 (57.7)	57 (42.5)	46 (25.4)	35 (18.9)	
Sex^a^								0.020
Male	635 (42.8)	173 (46.1)	119 (36.3)	113 (40.5)	70 (52.2)	82 (45.3)	78 (42.2)	
Female	847 (57.2)	202 (53.9)	209 (63.7)	166 (59.5)	64 (47.8)	99 (54.7)	107 (57.8)	
Higher education level^a^ ^,^^c^								<0.001
No	916 (63.0)	123 (32.9)	249 (75.9)	181 (64.9)	113 (89.7)	121 (70.3)	129 (74.1)	
Yes	537 (37.0)	251 (67.1)	79 (24.1)	98 (35.1)	13 (10.3)	51 (29.7)	45 (25.9)	
*Missing*	*29*	*1*	*0*	*0*	*8*	*9*	*11*	
Number of people in household^a^								<0.001
1	340 (23.5)	98 (26.1)	67 (20.6)	100 (36.2)	23 (18.3)	23 (13.5)	29 (16.7)	
2	402 (27.8)	165 (44.0)	93 (28.6)	68 (24.6)	27 (21.4)	31 (18.1)	18 (10.3)	
3	254 (17.6)	46 (12.3)	77 (23.7)	52 (18.8)	24 (19.0)	36 (21.1)	19 (10.9)	
4	262 (18.1)	55 (14.7)	58 (17.8)	37 (13.4)	27 (21.4)	45 (26.3)	40 (23.0)	
≥5	189 (13.1)	11 (2.9)	30 (9.2)	19 (6.9)	25 (19.8)	36 (21.1)	68 (39.1)	
*Missing*	*35*	*0*	*3*	*3*	*8*	*10*	*11*	
Cultural orientation^a^ ^,^^d^								<0.001
More integrated	1,282 (89.3)	375 (100.0)	283 (87.1)	246 (88.8)	98 (79.0)	138 (82.1)	142 (85.0)	
Less integrated	154 (10.7)	0 (0.0)	42 (12.9)	31 (11.2)	26 (21.0)	30 (17.9)	25 (15.0)	
*Missing*	*46*	*0*	*3*	*2*	*10*	*13*	*18*	
Health literacy^a^								<0.001
Adequate	1,344 (92.3)	373 (99.5)	317 (96.6)	275 (98.6)	91 (71.7)	136 (78.6)	152 (87.4)	
Low	112 (7.7)	2 (0.5)	11 (3.4)	4 (1.4)	36 (28.3)	37 (21.4)	22 (12.6)	
*Missing*	*26*	*0*	*0*	*0*	*7*	*8*	*8*	
Level of trust in the government pandemic response^e^								<0.001
Trust	634 (42.8)	178 (47.5)	126 (38.4)	97 (34.8)	105 (78.4)	60 (33.1)	68 (36.8)	
Neutral	650 (43.9)	151 (40.3)	172 (52.4)	138 (49.5)	22 (16.4)	74 (40.9)	93 (50.3)	
No trust	198 (13.4)	46 (12.3)	30 (9.1)	44 (15.8)	7 (5.2)	47 (26.0)	24 (13.0)	
Self-reported SARS-CoV-2 vaccination uptake (primary series)^e^ ^,^^f^								<0.001
Unvaccinated	195 (13.2)	16 (4.3)	25 (7.6)	59 (21.1)	6 (4.5)	33 (18.2)	56 (30.3)	
Incomplete primary series	5 (0.3)	0 (0.0)	1 (0.3)	1 (0.4)	2 (1.5)	0 (0.0)	1 (0.5)	
Complete primary series	1,282 (86.5)	359 (95.7)	302 (92.1)	219 (78.5)	126 (94.0)	148 (81.8)	128 (69.2)	
At least 1 dose	1,287 (86.8)	359 (95.7)	303 (92.4)	220 (78.9)	128 (95.5)	148 (81.8)	129 (69.7)	
Self-reported booster uptake, among those who completed the primary series^e^ ^,^^g^								<0.001
No	343 (26.8)	36 (10.0)	85 (28.1)	57 (26.0)	34 (27.0)	72 (48.6)	59 (46.1)	
Yes	939 (73.2)	323 (90.0)	217 (71.9)	162 (74.0)	92 (73.0)	76 (51.4)	69 (53.9)	
SARS-CoV-2 antibody test result at visit 3^e^								0.899
Negative	22 (1.5)	5 (1.3)	4 (1.2)	6 (2.2)	1 (0.7)	3 (1.7)	3 (1.6)	
Positive	1,460 (98.5)	370 (98.7)	324 (98.8)	273 (97.8)	133 (99.3)	178 (98.3)	182 (98.4)	
Infection and vaccination status among those seropositive at visit 3^e^ ^,^^h^								<0.001
Infected and vaccinated	794 (54.4)	215 (58.1)	170 (52.5)	122 (44.7)	78 (58.6)	117 (65.7)	92 (50.6)	
Only vaccinated	488 (33.4)	142 (38.4)	132 (40.7)	97 (35.5)	49 (36.8)	31 (17.4)	37 (20.3)	
Only infected	178 (12.2)	13 (3.5)	22 (6.8)	54 (19.8)	6 (4.5)	30 (16.9)	53 (29.1)	
Month of study visit 3 (in 2022)								<0.001
May	40 (2.7)	23 (6.1)	13 (4.0)	0 (0.0)	0 (0.0)	0 (0.0)	4 (2.2)	
June	314 (21.2)	138 (36.8)	90 (27.4)	40 (14.3)	0 (0.0)	0 (0.0)	46 (24.9)	
July	480 (32.4)	136 (36.3)	105 (32.0)	118 (42.3)	36 (26.9)	40 (22.1)	45 (24.3)	
August	304 (20.5)	35 (9.3)	66 (20.1)	73 (26.2)	54 (40.3)	47 (26.0)	29 (15.7)	
September	228 (15.4)	27 (7.2)	38 (11.6)	38 (13.6)	30 (22.4)	64 (35.4)	31 (16.8)	
October	73 (4.9)	8 (2.1)	8 (2.4)	7 (2.5)	9 (6.7)	20 (11.0)	21 (11.4)	
November	43 (2.9)	8 (2.1)	8 (2.4)	3 (1.1)	5 (3.7)	10 (5.5)	9 (4.9)	

Abbreviations: HELIUS, Healthy Life in an Urban Setting; COVID-19, Coronavirus disease 2019; IQR, interquartile range; SARS-CoV-2, severe acute respiratory syndrome coronavirus 2.

a Measured at HELIUS baseline (2011–2015).

b Age was recalculated for the third COVID-19 substudy visit.

c Higher education level includes higher vocational schooling and university; lower education level includes no/elementary school, lower/intermediate vocational schooling, lower/intermediate secondary school.

d Participants were classified as being more integrated into the host society when not applicable (Dutch ethnic origin) or when measured to be integrated or assimilated; participants were classified as less integrated when measured to be separated or marginalized, according to Berry’s acculturation strategies (reference: Berry JW. Immigration, acculturation, and adaptation. Applied Psychol: An International Review 1997; 46: 5–68).

e Measured during the third COVID-19 substudy visit (May to November 2022).

f SARS-CoV-2 vaccination status was determined by the question ‘Which primary vaccinations have you received?’. Incomplete: received one dose of a vaccine other than Janssen, with or without subsequent infection; complete: received two doses of Pfizer, Moderna or AstraZeneca, ≥1 dose of Janssen, or had a past infection and subsequently received ≥1 dose of any vaccine (based on the guidelines of the Dutch government, reference: National Institute for Public Health and the Environment. COVID-19-vaccinatie uitvoeringsrichtlijn – version 4 December 2021. 2021. Available from: https://lci.rivm.nl/richtlijnen/covid-19-vaccinatie. Accessed 20 March 2023).

g Booster status was determined by the question ‘Have you received a booster vaccination?’.

h Prior infection and vaccination status was defined as being only previously vaccinated (based on the self-reported uptake of ≥1 SARS-CoV-2 vaccine dose, without evidence of prior SARS-CoV-2 infection), only previously infected (based on having a positive antibody test result at the third COVID-19 substudy visit without reporting to be previously vaccinated), or both previously infected and vaccinated (based on the self-reported uptake of at least one SARS-CoV-2 vaccine dose and having tested seropositive during previous substudy visits (visit 1: June to October 2020 or visit 2: November 2020 to June 2021) or, if antibody test results during previous visit were negative or unavailable, on self-reported prior infection).

A total of 1,287 participants (86.8%) reported to have received at least one SARS-CoV-2 vaccine dose. Among them, 1,282 (99.6%) completed the primary series (i.e., two doses of Pfizer, Moderna or AstraZeneca, at least one dose of Janssen, or infection prior to receiving at least one dose of any vaccine), and of them, 939 (73.2%) received a booster dose. Self-reported vaccination uptake varied significantly between ethnic groups, with the proportion of participants who received at least one dose being highest in the Dutch (95.7%) and Ghanaian (95.5%) groups and lowest in the Moroccan group (69.7%). Among those who received at least one dose, the booster uptake was highest in the Dutch group (90.0%) and lowest in the Turkish (51.4%) and Moroccan (53.5%) groups.

### Prevalence of anti-spike SARS-CoV-2 antibodies

Of all analysed participants of the third COVID-19 substudy visit, 1,460 (98.5%) had SARS-CoV-2 spike protein antibodies at the time of their study visit between May and November 2022, while 22 (1.5%) did not (Table 1). The proportion of individuals with antibodies did not differ significantly between ethnic groups (*P* = 0.899). Most other participant characteristics were also similar between those with and without antibodies (Supplementary Table S2).

### Ethnic variation in SARS-CoV-2 infection and vaccination status

**Figure 1.**
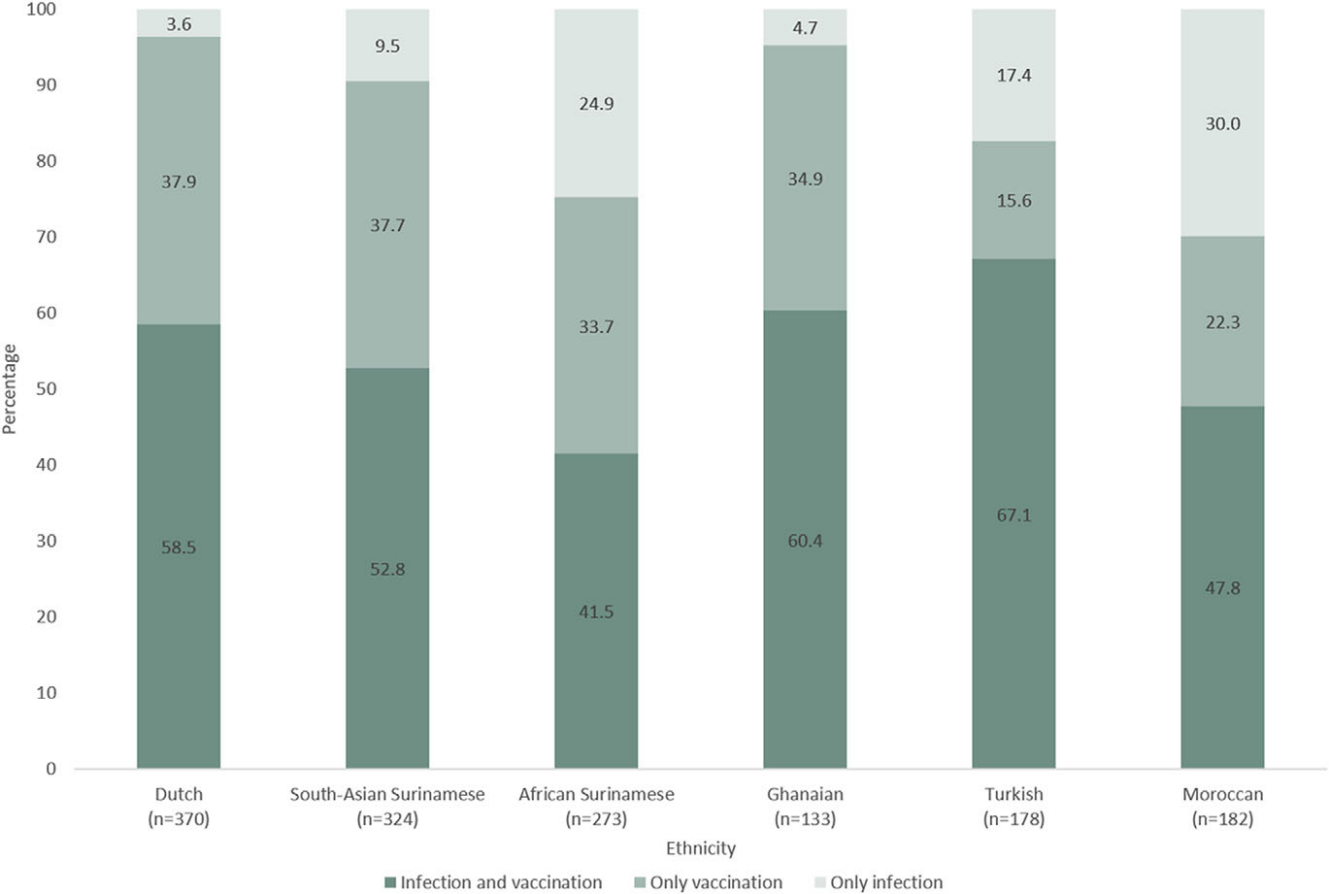
SARS-CoV-2 infection and vaccination status^a^ among HELIUS participants with a positive SARS-CoV-2 WANTAI ELISA antibody test result, per ethnic group, Amsterdam, the Netherlands, 17 May 2022 to 21 November 2022 (*n* = 1,460). Abbreviations: SARS-CoV-2, severe acute respiratory syndrome coronavirus 2; HELIUS, Healthy Life in an Urban Setting; ELISA, enzyme-linked immunosorbent assay. ^a^Prior infection and vaccination status was defined as being only previously vaccinated (based on the self-reported uptake of ≥1 SARS-CoV-2 vaccine dose, without evidence of prior SARS-CoV-2 infection), only previously infected (based on having a positive antibody test result at the third COVID-19 substudy visit without reporting to be previously vaccinated), or both previously infected and vaccinated. Among the 794 participants who had both prior infection and vaccination, prior infection was determined based on a positive antibody test result during the second substudy visit (November 2020 to June 2021) (*n* = 264), or during the first substudy visit when the test result from the second visit was unavailable (June to October 2020) (*n* = 5). For cases where previous antibody test results were negative or missing, prior infection was based on self-report, which varied between the first and third (*n* = 36) and second and third visit (*n* = 489), depending on the last substudy visit during which the participant tested seronegative. The infection and vaccination status estimates account for the age and sex distribution of the Amsterdam population through post-stratification weights and for sampling. The uncorrected and corrected estimates and their 95% confidence intervals are shown in Supplementary Figure S3.

Of the 1,460 participants with SARS-CoV-2 antibodies, 54.4% were both previously infected and vaccinated (*n* = 794), 33.4% were only previously vaccinated (*n* = 488), and 12.2% were only previously infected (*n* = 178). The distribution of infection and vaccination status differed significantly between ethnic groups (*P* < 0.001) (Table 1). Being previously infected and vaccinated was most common in the Turkish (corrected percentage accounting for the population structure of Amsterdam and sampling 67.1%, 95% CI = 59.1–75.0%), followed by the Ghanaian (60.4%, 95% CI = 51.2–69.6%), Dutch (58.5%, 95% CI = 53.1–63.8), South-Asian Surinamese (52.8%, 95% CI = 46.8–58.9%), Moroccan (47.8%, 95%CI = 39.7–55.9%), and African Surinamese (41.5%, 95% CI = 35.0–47.9%) groups (Figure 1, uncorrected and corrected estimates and corresponding 95% CI can be found in Supplementary Figure S3). Being only previously vaccinated was least common in the Turkish (15.6%, 95% CI = 9.7–21.5%) and most common in the Dutch (37.9%, 95% CI = 32.7–43.1%) group. Being only previously infected varied between 3.6% (95% CI = 1.7–5.6%) in the Dutch and 30.0% (95% CI = 22.6–37.3%) in the Moroccan group.

**Figure 2.**
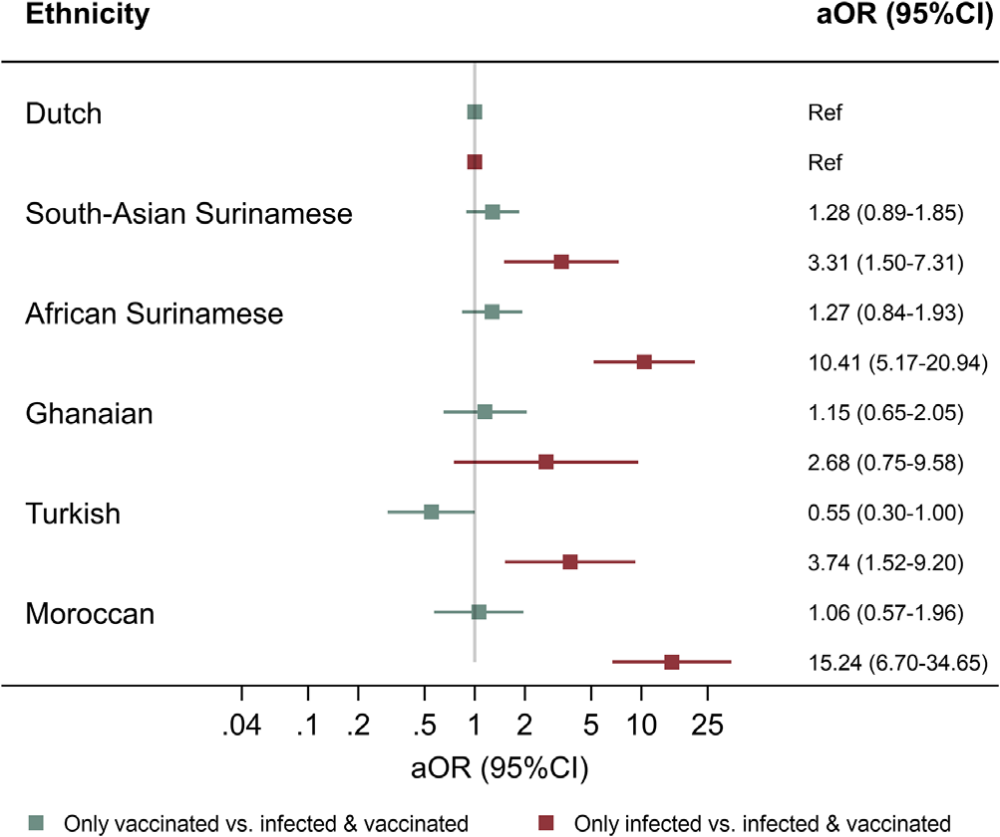
Ethnic variation in SARS-CoV-2 vaccination and infection status^a^ among HELIUS participants with a positive SARS-CoV-2 antibody test result, Amsterdam, the Netherlands, 17 May 2022 to 21 November 2022.^b^ Abbreviations: SARS-CoV-2, severe acute respiratory syndrome coronavirus 2; HELIUS, Healthy Life in an Urban Setting; aOR, adjusted odds ratio; CI, confidence interval; Ref, reference category. ^a^Prior infection and vaccination status was defined as being only previously vaccinated (based on the self-reported uptake of ≥1 SARS-CoV-2 vaccine dose, without evidence of prior SARS-CoV-2 infection), only previously infected (based on having a positive antibody test result at the third COVID-19 substudy visit without reporting to be previously vaccinated), or both previously infected and vaccinated (based on the self-reported uptake of at least one SARS-CoV-2 vaccine dose and having tested seropositive during previous substudy visits [visit 1: June to October 2020 or visit 2: November 2020 to June 2021] or, if antibody test results during previous visit were negative or unavailable, on self-reported prior infection). ^b^Analyses were performed using multinomial logistic regression (reference = both infected and vaccinated). Observations with missing values on covariates were removed from analysis. The model is adjusted for age, sex, household size, trust in the government response in containing the SARS-CoV-2 pandemic, and month of study visit. Analyses account for sampling and for the age and sex distribution of the Amsterdam population through post-stratification weights.

In both univariable analysis and the analysis adjusted for age, sex, household size, trust in the government’s response to the pandemic, and month of study visit (model 1), participants of South-Asian Surinamese (adjusted odds ratio (aOR) = 3.31, 95% CI = 1.50–7.31)), African Surinamese (aOR = 10.41, 95% CI = 5.17–20.94), Turkish (aOR = 3.74, 95%CI = 1.52–9.20), or Moroccan (aOR = 15.24, 95%CI = 6.70–34.65) origin were significantly more likely to be only infected than both infected and vaccinated, compared to participants of Dutch origin (Figure 2, Supplementary Table S3). These associations remained similar when only including individuals with a SARS-CoV-2 antibody test result at all three substudy visits (Supplementary Table S4). No significant differences were observed between ethnic groups for being only vaccinated versus both infected and vaccinated.

In multivariable analysis (model 1), alongside ethnicity, age and level of trust in the Dutch government’s response in containing the pandemic were significantly associated with SARS-CoV-2 infection and vaccination status (Supplementary Table S3). Participants who were neutral about or had no trust in the government’s response were more likely to be only infected than both infected and vaccinated, compared to those trusting the government’s response.

After additionally adjusting for cultural orientation and health literacy, while excluding the Dutch group (model 2), individuals of African Surinamese or Moroccan origin were more likely to be only infected, and individuals of Turkish origin were less likely to be only vaccinated, than infected and vaccinated, compared to those of South-Asian Surinamese origin (Supplementary Table S3).

Based on the E-value analysis, the association of the unmeasured confounder with both ethnicity and particularly prior infection (versus both infection and vaccination) would need to be strong to explain away the current effect (Supplementary Table S5).

## Discussion

This analysis of an adult multi-ethnic population-based cohort in Amsterdam, the Netherlands, demonstrated that 98.5% of the individuals had developed antibodies against SARS-CoV-2 in the second half of 2022. Notwithstanding the lack of differences in SARS-CoV-2 antibody prevalence between ethnic groups, our analyses did reveal ethnic differences in the combination of prior SARS-CoV-2 infection and vaccination among those with antibodies in the second half of 2022. Being both previously infected and vaccinated against SARS-CoV-2 was most common in the Turkish group (67%), followed by the Ghanaian (60%), Dutch (59%), South-Asian Surinamese (53%), Moroccan (48%), and African Surinamese (42%) groups. When comparing to individuals with both prior infection and vaccination, and after accounting for age, sex, household size, trust in the government’s response to the pandemic, and month of study visit, individuals of South-Asian Surinamese, African Surinamese, Turkish, or Moroccan origin were more likely to be only infected versus those of Dutch origin.

The prevalence of anti-spike SARS-CoV-2 antibodies was high and similar among the studied ethnic groups. This result might seem unexpected, given that the cumulative incidence of SARS-CoV-2 infections varied significantly between ethnic groups in Amsterdam by 31 March 2021 [5]. However, by mid-2022, much of the Dutch population had been infected with SARS-CoV-2, partly due to the highly transmissible Omicron variant, which became dominant in December 2021 [7, 20], and the abolishment of mitigation measures [21]. Furthermore, the entire population had the opportunity to receive a primary vaccination, and in November 2021, a nationwide booster vaccination campaign was implemented [21]. These events likely led to a large increase in the SARS-CoV-2 antibody prevalence. In line with our findings, 98% of Dutch blood donors had natural or vaccine-induced antibodies against SARS-CoV-2 by February 2022, though this study was unable to compare between ethnic groups [22]. Despite the high prevalence of anti-spike SARS-CoV-2 antibodies among our participants, 1.5% lacked antibodies, emphasizing the ongoing need for intervention efforts to protect these people against infection and severe disease progression. Addressing factors, such as lack of trust in the government’s response to the pandemic, which appeared to be lower among those lacking antibodies, could help enhance vaccination uptake.

The prior SARS-CoV-2 infection and vaccination status varied remarkably between ethnic groups. First, we observed that 30%, 25%, 17%, and 10% of the individuals of Moroccan, African Surinamese, Turkish, and South-Asian Surinamese origin, respectively, were only previously infected without vaccination, compared to only 4% of those of Dutch origin. The differences remained when adjusting for age, sex, household size, trust in the government’s response to the pandemic, and month of study visit. These observations align with prior research, in which linkage of SARS-CoV-2 vaccination registry data to HELIUS data demonstrated lower vaccination uptake among these groups, except in the South-Asian Surinamese group, between January and September 2021 [6]. Ethnic minority groups, and especially those unvaccinated, face a higher risk of developing severe COVID-19-related outcomes following infection, emphasizing the importance of vaccination in these groups [23]. However, ethnic minority groups have experienced hesitancy towards SARS-CoV-2 vaccination, driven by underlying structural disadvantages (e.g., geographical, economic, social), concerns about vaccine effectiveness and safety, language barriers, culture, mistrust in the government and health systems, and misinformation [6, 16, 24, 25]. In response to practical barriers, the Public Health Service of Amsterdam has implemented tailored interventions to encourage vaccination uptake, including collaborating with community leaders, providing information in native languages, and deploying an increasing amount of mobile vaccination units across city districts. Data on practical barriers (e.g., distance to vaccination location) was unavailable for our analyses, but merits further investigation. Nevertheless, the E-value analysis suggests that unmeasured variables would have to be highly confounding to change the identified associations. As factors related to vaccination intent and uptake for SARS-CoV-2, but also other infectious diseases, can be specific to certain ethnic groups [6, 16], tailored strategies addressing these concerns are crucial.

Our findings revealed that the slight majority of participants had acquired immunity through both prior SARS-CoV-2 infection and vaccination, varying between 67% in the Turkish group and 42% in the African Surinamese group. A combination of antibodies acquired through both prior SARS-CoV-2 infection and vaccination (i.e., hybrid immunity) offers more protection against SARS-CoV-2 infection and severe disease progression than natural or vaccine-induced immunity alone [9, 26, 27]. Findings from a systematic review and meta-analysis additionally suggested that hybrid immunity offers longer lasting protection against reinfection compared to either infection or, to a larger extent, vaccination alone [9]. However, concerns persist regarding waning immunity and the potential for antibody evasion by emerging SARS-CoV-2 variants [28], emphasizing the ongoing importance of vaccination, even following infection. It is, however, important that vaccination precedes infection, as infection could lead to severe COVID-19, a risk reduced by vaccination [23, 29]. Concerningly, there appears to be a higher risk of infection preceding vaccination in ethnic minority groups, assumed by the higher incidence of SARS-CoV-2 infections compared to the Dutch origin group in the pre-vaccination era [5]. Consequently, these groups had been at increased risk of severe outcomes associated with infection, such as COVID-19-related hospitalization, ICU admission, mortality, and developing post-COVID-19 complications [1].

The prior SARS-CoV-2 infection and vaccination status across ethnic groups had not previously been investigated in the Netherlands. However, a study from the United States (US) demonstrated variation in the prior infection and vaccination status between ethnic groups, with hybrid immunity ranging between 26.5% among Hispanic and 15.4% among Asian individuals [30]. It should be noted that the US study was conducted when the Delta variant was dominantly circulating (i.e., January and December 2021), and ethnic backgrounds and cultural histories of ethnic groups vary between the Netherlands and the US.

This study has several limitations. First, there is a potential for misclassification of SARS-CoV-2 infection or vaccination status. The WANTAI SARS-CoV-2 antibody ELISA does not discriminate between antibodies acquired through infection or vaccination, as it measures spike protein antibodies, indicating prior infection or vaccination, and not nucleocapsid protein antibodies, which specifically indicate prior infection. Hence, we partly relied on self-report for determining the infection and vaccination status. The number of vaccinated individuals might have been overestimated, as participants potentially provided socially desirable answers regarding their vaccination status. However, the high uptake of 87% by November 2022 was consistent with national vaccination data (82% of the population ≥18 years old and 94% of those ≥60 years old in the Netherlands had received at least one dose by the end of 2022 [31]). Additionally, the differences in SARS-CoV-2 vaccination uptake we observed between ethnic groups align with previous findings from the HELIUS cohort, based on registry data from September 2021 [6]. Self-reported prior infections might have been overestimated, as some participants were classified as previously infected regardless of whether these infections were suspected or confirmed, or underestimated, as participants might have had asymptomatic infections. Infections that passed mostly unnoticed were more common in the Ghanaian group compared to other ethnic groups within the HELIUS cohort [4], potentially leading to an overestimation of participants classified as only vaccinated in this group. It should be noted that it is uncertain whether individuals with prior infection, vaccination, or both were still protected against COVID-19 at the time of their study visit, as antibody levels might have declined over time, even in individuals with hybrid immunity, potentially reducing the level of protection [9]. Furthermore, the sociodemographic and cultural differences between participants in the COVID-19 substudy and the parent HELIUS cohort suggest potential selection bias. Given the higher proportions of individuals with factors that might be associated with increased vaccination uptake and lower infection risk (e.g., more highly educated, higher health literacy), this bias could have led towards higher vaccination and lower infection rates. However, since the numeric differences in percentages between included and non-included individuals were not noteworthy, this bias was likely limited. Lastly, changes may have occurred in the measured household size, cultural orientation, and health literacy since the baseline visit of the HELIUS study (i.e., 2011–2015), which might not have been fully representative of their values at time of measurement of our study outcomes in 2022.

In conclusion, while seroprevalence was high and similar across the studied ethnic groups, the acquisition of SARS-CoV-2 spike protein antibodies (i.e., naturally, through immunization, or both) varied between the groups, notably with a higher proportion of individuals in the Moroccan, African Surinamese, Turkish, and South-Asian Surinamese groups having acquired antibodies only through previous infection compared to the Dutch group. As hybrid immunity offers greater protection than natural or vaccine-induced immunity alone, our findings could help guide policy makers in prioritizing future vaccination and booster campaigns for specific demographic groups, such as those only previously infected. As governmental mistrust was associated with a higher likelihood of being only infected without vaccination, exploring strategies to overcome this mistrust is essential for enhancing future uptake of vaccination against SARS-CoV-2 and other infectious diseases.

## Supporting information

Campman et al. supplementary material Campman et al. supplementary material

## Data Availability

Data requests can be submitted to the steering committee of the HELIUS study.

## Supplementary material

For supplementary material accompanying this paper visit http://doi.org/10.1017/S0950268825000056. click here to view supplementary material
