# SARS-CoV-2 infection and vaccination status in six ethnic groups in Amsterdam, The Netherlands, May to November 2022 – CORRIGENDUM

**DOI:** 10.1017/S0950268825000342

**Published:** 2025-03-31

**Authors:** Sophie L. Campman, Anders Boyd, Janke Schinkel, Liza Coyer, Charles Agyemang, Henrike Galenkamp, Anitra D.M. Koopman, Felix P. Chilunga, Jelle Koopsen, Aeilko H. Zwinderman, Suzanne Jurriaans, Karien Stronks, Maria Prins

**Affiliations:** 1 Department of Infectious Diseases, Public Health Service of Amsterdam, Amsterdam, The Netherlands; 2 Amsterdam UMC location University of Amsterdam, Infectious Diseases, Amsterdam, The Netherlands; 3 Amsterdam Institute for Immunology and Infectious Diseases, Amsterdam, The Netherlands; 4 Stichting HIV Monitoring, Amsterdam, The Netherlands; 5 Amsterdam UMC location AMC, University of Amsterdam, Department of Medical Microbiology and Infection Prevention, Amsterdam, The Netherlands; 6 Department of Public and Occupational Health, Amsterdam Public Health Research Institute, Amsterdam UMC, University of Amsterdam, Amsterdam, The Netherlands; 7 Department of Medicine, Division of Endocrinology, Diabetes and Metabolism, Johns Hopkins University School of Medicine, Baltimore, MA, USA; 8 Amsterdam Public Health, Health Behaviors and Chronic Diseases, Amsterdam, The Netherlands; 9 Amsterdam UMC location University of Amsterdam, Clinical Epidemiology, Biostatistics and Bioinformatics, Amsterdam, The Netherlands

When this article was originally published in Epidemiology and Infection it contained errors in the STATA syntax. In the analyses, the authors appliedpost-stratification weights to account for the age and sex distribution within ethnic groups in the Amsterdam population. However, these weights were not correctly applied, affecting some of the reported results.

After correctly applying the post-stratification weights, Figures 1 and 2, as well as Figure S3 and Tables S3, S4 and S5 in the Supplementary materials have slightly changed, along with the corresponding text in the article.

While the conclusions of the study remain unchanged after these updates, the authors believe it is important that these were corrected to ensure accuracy and transparency in their research.

The authors apologise for these errors.
