# Supplementary material for: SARS-CoV-2 infection and vaccination status in six ethnic groups in Amsterdam, The Netherlands, May to November 2022
Source: Epidemiol Infect. 2025 Jan 23;153:e23. doi: 10.1017/S0950268825000056 (PMC11822580; doi:10.1017/S0950268825000056)
Supplement: Campman et al. supplementary material [file S0950268825000056sup001.docx]

**Supplementary materials to:**

**SARS-CoV-2 infection and vaccination status in six ethnic groups in Amsterdam, the Netherlands,** **May-November 2022**

**Authors**

Sophie L. Campman, Anders Boyd, Janke Schinkel, Liza Coyer, Charles Agyemang, Henrike Galenkamp, Anitra D.M. Koopman, Felix P. Chilunga, Jelle Koopsen, Aeilko H. Zwinderman, Suzanne Jurriaans, Karien Stronks, Maria Prins.

**Contents**

**Supplementary Methods 1**. Categorization of prior SARS-CoV-2 infection and vaccination status among HELIUS participants with a positive SARS-CoV-2 WANTAI ELISA antibody test result at the third COVID-19 substudy visit, Amsterdam, the Netherlands, May 17, 2022 - November 21, 2022 (n=1,460).

**Supplementary Figure S1**. Schematic representation of determining prior infection among HELIUS participants with a positive SARS-CoV-2 WANTAI ELISA antibody test result at the third COVID-19 substudy visit who received at least one SARS-CoV-2 vaccine dose (n=794).

**Supplementary Methods 2**. Sensitivity analysis.

**Supplementary Methods 3**. Information on correcting for sampling and post-stratification.

**Supplementary Figure S2**. Flowchart of the inclusion of HELIUS participants in the third visit of the longitudinal COVID-19 substudy, Amsterdam, the Netherlands, May 17 - November 21, 2022.

**Supplementary Table S1.** Characteristics of HELIUS participants included in the baseline study and COVID-19 substudy visit 3, Amsterdam, the Netherlands, May 17 - November 21, 2022.

**Supplementary Table S2**. Characteristics of HELIUS participants who tested positive or negative for antibodies against SARS-CoV-2, Amsterdam, the Netherlands, May 17, 2022 - November 21, 2022 (n=1,482).

**Supplementary Figure S3**. Uncorrected and corrected SARS-CoV-2 infection and vaccination status among HELIUS participants with a positive SARS-CoV-2 WANTAI ELISA antibody test result, per ethnic group, Amsterdam, the Netherlands, May 17, 2022 - November 21, 2022 (n=1,460).

**Supplementary Table S3.** Association between ethnicity and SARS-CoV-2 vaccination and infection status, while accounting for sociodemographic, access to healthcare, and acculturation determinants, among HELIUS participants with a positive SARS-CoV-2 WANTAI ELISA antibody test result, Amsterdam, the Netherlands, May 17, 2022 - November 21, 2022.

**Supplementary Table S4**. Association between ethnicity and SARS-CoV-2 vaccination and infection status among HELIUS participants with a positive SARS-CoV-2 WANTAI ELISA antibody test result who had antibody test results from all three COVID-19 substudy visits, Amsterdam, the Netherlands, May 17, 2022 - November 21, 2022 (sensitivity analysis).

**Supplementary Table S5.** E-value analysis assessing the minimum strength of association of unmeasured confounders needed to explain the observed association between ethnicity and SARS-CoV-2 infection and vaccination status.

**Supplementary Methods 1.** Categorization of prior SARS-CoV-2 infection and vaccination status among HELIUS participants with a positive SARS-CoV-2 WANTAI ELISA antibody test result at the third COVID-19 substudy visit, Amsterdam, the Netherlands, May 17, 2022 - November 21, 2022 (n=1,460).

1. **Only prior vaccination** was defined as testing positive for SARS-CoV-2 antibodies during the third COVID-19 substudy visit, measured using WANTAI ELISA, and reporting to have previously received at least one vaccine dose against SARS-CoV-2, while not testing positive for SARS-CoV-2 antibodies during previous COVID-19 substudy visits [visit 1: June-October 2020 or visit 2: November 2020-June 2021], nor self-reporting previous infection since the last negative antibody test result. Previous vaccination was determined by the question, “Have you received a COVID-19 vaccination?” (i.e., at least one dose), which was asked during the third substudy visit, and defined as having received at least one vaccine dose against SARS-CoV-2. A total of 488 participants were categorized as only previously vaccinated in analysis.
2. **Only prior infection** was defined as testing positive for SARS-CoV-2 antibodies during the third substudy visit, measured using WANTAI ELISA, while reporting not to be previously vaccinated. A total of 178 participants were categorized as only previously infected in analysis.
3. **Both prior infection and vaccination** was determined as schematically described in Supplementary Figure S1. Prior infection and vaccination was defined as testing positive for SARS-CoV-2 antibodies during the third substudy visit, measured using WANTAI ELISA, while reporting to have previously received at least one vaccine dose against SARS-CoV-2, and while testing positive for SARS-CoV-2 antibodies during the second substudy visit (November 2020-June 2021) (3a), or the first substudy visit (June-October 2020) if the test result from the second visit was unavailable (3b). We excluded the few participants who reported receiving vaccination before the second substudy visit from analysis (n=6), and we assumed that seropositivity during these previous substudy visits resulted from infection as vaccination against SARS-CoV-2 was largely unavailable ^1^. When previous SARS-CoV-2 antibody test results were negative or missing, prior SARS-CoV-2 infection was determined based on self-report at the third substudy visit, determined by the question, “Have you had a coronavirus infection since the start of the coronavirus pandemic, i.e., since the beginning of 2020?”. This question included both confirmed (i.e., through rapid antigen test or Nucleic Acid Amplification Test by a health professional or rapid antigen self-test) and suspected (i.e., not confirmed by any test) infections. Self-reported prior infection could have occurred between the second and third (3c) and first and third visit (3d), depending on the last substudy visit at which the participant tested seronegative. If antibody test results were unavailable for both the first and second visits, we would base infection on self-reported infection between the start of the pandemic and third visit. We used the self-reported month and year infection to determine whether participants were infected between the start of the pandemic and the third, first and third, or second and third visit. This way, we only included infections that were reported since the last substudy visit during which the participant tested seronegative (usually the second substudy visit), reducing the risk of including false self-reported infections. A total of 794 participants were categorized as both previously infected and vaccinated in analysis.

^1^ National Institute for Public Health and the Environment (RIVM). Deelname COVID-19-vaccinatie in Nederland. 2021. Available from: . Accessed on: 23 January 2024.

Ab+

Ab+

Ab+/Ab-/missing

Ab+ at visit 2 (n=264)

**3a**

Ab+

**3b**

Ab+

Ab+ at visit 1 (n=5)

Missing

Ab+

**3c**

Self-reported prior infection between visit 2 and 3 (n=489)

Ab-

Ab-/missing

Ab+

**3d**

Ab+

Self-reported prior infection between visit 1 and 3 (n=36)

Missing

Ab-

**3e**

Self-reported prior infection between start of the pandemic and visit 3 (n=0)

Ab+

Missing

Missing

**Supplementary Figure S1**. Schematic representation of determining prior infection among HELIUS participants with a positive SARS-CoV-2 WANTAI ELISA antibody test result at the third COVID-19 substudy visit who received at least one SARS-CoV-2 vaccine dose (n=794). Abbreviations: *HELIUS* Healthy Life in an Urban Setting; *SARS-CoV-2* Severe Acute Respiratory Syndrome Coronavirus 2; *ELISA* enzyme-linked immunosorbent assay; *COVID-19* Coronavirus disease 2019; *Ab* antibody.

**Supplementary Methods 2.** Sensitivity analysis.

Individuals who participated in earlier substudy visits, but did not have an antibody test result at subsequent visits, did not have the same testing probability over time compared to those with antibody testing results at all three substudy visits, and thus could have biased estimates. Therefore, in sensitivity analysis, we repeated our analyses only including individuals who had a SARS-CoV-2 antibody test result for all three COVID-19 substudy visits.

**Supplementary Methods 3.** Information on correcting for sampling and post-stratification.

For sampling, the probability of being invited for the COVID-19 substudy (among those in active follow-up in the parent HELIUS study), the conditional probability of participating in the first COVID-19 substudy visit (among those invited, given the participant’s ethnicity, age, educational level, working status, and health literacy), and the conditional probability of participating in the third substudy visit (among those participating in the first study visit, given the participant’s ethnicity, age and educational level) were calculated. The inverse of the product of these probabilities was taken, standardized to one, and used as a sampling weight. For post-stratification, a weight was assigned corresponding to the proportion representing the Amsterdam population of each stratum of age (20-44, 45-54, 55-59, ≥60 years), sex (male, female) and ethnicity (Dutch, Surinamese, Ghanaian, Turkish, Moroccan). Sampling and post-stratification weights were used in line with the ’svy’ commands in STATA.

**HELIUS baseline participants, randomly selected from the municipality registry, included between 2011 and 2015**

n=24,780

**Excluded (n=7,934)**

- Deceased or moved abroad
- No consent to be re-invited
- Current zip code unknown, outside of Amsterdam, or too far from HELIUS study locations (due to COVID-19 measures, travel time was kept limited)
- Recently participated in or invited to another HELIUS substudy
- Ethnic background other than Dutch, South-Asian Surinamese, African Surinamese, Ghanaian, Turkish, or Moroccan

**HELIUS participants eligible for participation in the COVID-19 substudy**
n=16,846 (68.0%)

**Participants invited for the COVID-19 substudy**
n=11,078 (65.8%)

**Excluded (n=8,581)**

- No response or refused to participate

**Participants with a first COVID-19 substudy visit (June 24-October 9, 2020) and invited for the second and third visit**
n=2,496 (22.5%)

**Excluded (n=991)**

- Participants who did not attend the third substudy visit

**Participants with a second COVID-19 substudy visit (November 23, 2020-June 4, 2021)**
n=2,088 (83.7%)

**Participants with a third COVID-19 substudy visit (May 17-November 21, 2022)**
n=1,505 (60.3%)

**Excluded (n=23)**

- Participants without an antibody test result at the third substudy visit

n=13 (0.9%)

- Participants who received SARS-CoV-2 vaccination before the second substudy visit

n=6 (0.4%)

- Participants who had no data on self-reported vaccination status

n=1 (0.1%)

- Participants without date of self-reported prior infection

n=3 (0.2%)

**Participants included in analyses**
n=1,482 (98.5%)

**Figure 1.** Flowchart of the inclusion of HELIUS participants in the second visit of the longitudinal COVID-19 substudy, Amsterdam, the Netherlands, November 23, 2020 – March 31, 2021.

**Supplementary Figure S2**. Flowchart of the inclusion of HELIUS participants in the third visit of the longitudinal COVID-19 substudy, Amsterdam, the Netherlands, May 17 - November 21, 2022. Abbreviations: *HELIUS* Healthy Life in an Urban Setting; *COVID-19* Coronavirus disease 2019; *SARS-CoV-2* Severe Acute Respiratory Syndrome Coronavirus 2. In the first COVID-19 substudy visit, 2,496 individuals participated, of whom 2,088/2,496 attended the second visit and 1,505/2,496 attended the third visit. A total of 1,418 individuals participated in all three substudy visits. We excluded individuals who did not participate in the third substudy visit (*n*=991) from the analyses, as well as participants who had no antibody test result available at the third substudy visit (*n*=13), participants who reported to have received SARS-CoV-2 vaccination before the second substudy visit (*n*=6), and participants who lacked data on the SARS-CoV-2 infection and vaccination status [i.e., had no data on self-reported vaccination history (*n*=1) or date of self-reported prior infection among those without a previous positive SARS-CoV-2 antibody test result (*n*=3)]. In total, 1,482 participants who participated in the third substudy visit between May and November 2022 were included in analyses.

**Supplementary Table S1.** Characteristics of HELIUS participants included in the baseline study and COVID-19 substudy visit 3, Amsterdam, the Netherlands, May 17 - November 21, 2022.

To assess potential selection bias among COVID-19 substudy participants compared to the parent HELIUS cohort, which is representative for the population of Amsterdam, sociodemographic, access to healthcare, and cultural indicators were compared between those included and those not included in the third substudy visit. The Pearson’s χ^2^ test was used for categorical data, and the Kruskal-Wallis rank test for continuous data.

| **Characteristic** * | **Parent HELIUS cohort (n=23,927)** † | **HELIUS participants in COVID-19 substudy visit 3 (n=1,505)** | **HELIUS participants not in COVID-19 substudy visit 3 (n=22,422)** | **HELIUS participants included vs. not included in visit 3** ‡ |
| --- | --- | --- | --- | --- |
|  | n(%) | n(%) | n(%) | *P* value |
| **Ethnicity** |  |  |  | <0.001 |
| Dutch | 4,671 (19.5) | 380 (25.2) | 4,291 (19.1) |  |
| South-Asian Surinamese | 3,366 (15.1) | 332 (22.1) | 3,034 (13.5) |  |
| African Surinamese | 4,458 (18.6) | 285 (18.9) | 4,173 (18.6) |  |
| Ghanaian | 2,733 (11.4) | 136 (9.0) | 2,597 (11.6) |  |
| Turkish | 4,198 (17.6) | 186 (12.4) | 4,012 (17.9) |  |
| Moroccan | 4,501 (18.8) | 186 (12.4) | 4,315 (19.2) |  |
| **Age on August 1, 2022, in years, median (IQR)** | 54.0 (41.8-63.0) | 57.8 (48.5-65.2) | 53.7 (41.4-62.8) | <0.001 |
| *Missing* | *1,734* | *10* | *1,724* |  |
| **Sex** |  |  |  | 0.999 |
| Male | 10,207 (42.7) | 642 (42.7) | 9,565 (42.7) |  |
| Female | 13,720 (57.3) | 863 (57.3) | 12,857 (57.3) |  |
| **Higher educational level** ^a^ |  |  |  | <0.001 |
| No | 17,142 (74.1) | 933 (63.2) | 16,209 (74.8) |  |
| Yes | 6,000 (25.9) | 543 (36.8) | 5,457 (25.2) |  |
| *Missing* | *785* | *29* | *756* |  |
| **Number of people in household** |  |  |  | <0.001 |
| 1 | 4,727 (20.5) | 514 (21.5) | 4,380 (20.3) |  |
| 2 | 5,523 (24.0) | 624 (26.1) | 5,115 (23.7) |  |
| 3 | 4,329 (18.8) | 427 (17.8) | 4,068 (18.9) |  |
| 4 | 4,406 (19.1) | 468 (19.6) | 4,143 (19.2) |  |
| ≥5 | 4,039 (17.5) | 360 (15.0) | 3,848 (17.9) |  |
| *Missing* | *903* | *35* | *868* |  |
| **Cultural orientation** ^b^ ** |  |  |  | <0.001 |
| More integrated | 14,807 (80.6) | 921 (85.4) | 13,886 (80.3) |  |
| Less integrated | 3,556 (19.4) | 157 (14.6) | 3,399 (19.7) |  |
| *Missing* | *893* | *47* | *846* |  |
| **Health literacy** |  |  |  | <0.001 |
| Adequate | 19,758 (85.0) | 1,363 (92.2) | 18,395 (84.5) |  |
| Low | 3,486 (15.0) | 116 (7.8) | 3,370 (15.5) |  |
| *Missing* | *683* | *26* | *657* |  |
| **Difficulty with Dutch language** ** |  |  |  | <0.001 |
| No | 11,011 (59.3) | 728 (66.4) | 10,283 (58.8) |  |
| Yes | 7,571 (40.7) | 368 (33.6) | 7,203 (41.2) |  |
| *Missing* | *674* | *29* | *645* |  |

Abbreviations: *HELIUS* Healthy Life in an Urban Setting; *COVID-19* Coronavirus disease 2019; *IQR* interquartile range. † Excluding participants not belonging to one of the six ethnic groups included in the COVID-19 study. ‡ Pearson's χ^2^ or Wilcoxon rank-sum test, as appropriate. * Measured at baseline (2011-2015). Information on the questionnaires used has been previously described (Campman et al. SARS-CoV-2 vaccination uptake in six ethnic groups living in Amsterdam, the Netherlands: a registry-based study within the HELIUS cohort. Prev. Med., 2024, 178: 107822). ** Excluding the Dutch origin group. ^a^ Higher education level includes higher vocational schooling and university. ^b^ Participants were classified as being more integrated into the host society when measured to be integrated or assimilated; participants were classified as less integrated when measured to be separated or marginalized.

**Supplementary Table S2**. Characteristics of HELIUS participants who tested positive or negative for antibodies against SARS-CoV-2, Amsterdam, the Netherlands, May 17, 2022 - November 21, 2022 (n=1,482).

| **Characteristic** | **Negative antibody test result**  **(n=22)** | **Positive antibody test result (n=1,460)** |  |
| --- | --- | --- | --- |
|  | n (%) | n (%) | *P* value |
| **Ethnicity** ^a^ |  |  | 0.899 |
| Dutch | 5 (22.7) | 370 (25.3) |  |
| South-Asian Surinamese | 4 (18.2) | 324 (22.2) |  |
| African Surinamese | 6 (27.3) | 273 (18.7) |  |
| Ghanaian | 1 (4.5) | 133 (9.1) |  |
| Turkish | 3 (13.6) | 178 (12.2) |  |
| Moroccan | 3 (13.6) | 182 (12.5) |  |
| **Age in years**, median (IQR) ^ab^ | 52.5 (41.0-60.0) | 58.0 (49.0-65.0) | 0.035 |
| **Sex** ^a^ |  |  | 0.137 |
| Male | 6 (27.3) | 629 (43.1) |  |
| Female | 16 (72.7) | 831 (56.9) |  |
| **Higher education level** ^ac^ |  |  | 0.954 |
| No | 14 (63.6) | 902 (63.0) |  |
| Yes | 8 (36.4) | 529 (37.0) |  |
| *Missing* | *0* | *29* |  |
| **Number of people in household** ^a^ |  |  | 0.606 |
| 1 | 7 (31.8) | 333 (23.4) |  |
| 2 | 8 (36.4) | 394 (27.6) |  |
| 3 | 3 (13.6) | 251 (17.6) |  |
| 4 | 2 (9.1) | 260 (18.2) |  |
| ≥5 | 2 (9.1) | 187 (13.1) |  |
| *Missing* | *0* | *35* |  |
| **Cultural orientation** ^ad^ |  |  | 0.214 |
| More integrated | 17 (81.0) | 1,265 (89.4) |  |
| Less integrated | 4 (19.0) | 150 (10.6) |  |
| *Missing* | *1* | *45* |  |
| **Health literacy** |  |  | 0.172 |
| Adequate | 22 (100.0) | 1,322 (92.2) |  |
| Low | 0 (0.0) | 112 (7.8) |  |
| *Missing* | *0* | *26* |  |
| **Level of trust in the government pandemic response** ^e^ |  |  | <0.001 |
| Trust | 3 (13.6) | 631 (43.2) |  |
| Neutral | 10 (45.5) | 640 (43.8) |  |
| No trust | 9 (40.9) | 189 (12.9) |  |
| **Self-reported vaccination uptake** ^ef^ |  |  | <0.001 |
| No | 17 (77.3) | 178 (12.2) |  |
| Incomplete primary series | 0 (0.0) | 5 (0.3) |  |
| Complete primary series | 5 (22.7) | 1,277 (87.5) |  |
| **Self-reported booster uptake, among those who completed the primary series** ^eg^ |  |  | 0.503 |
| No | 2 (40.0) | 341 (26.7) |  |
| Yes | 3 (60.0) | 936 (73.3) |  |
| **Previous positive SARS-CoV-2 antibody test result (visit 1 or 2)** ^h^ |  |  | 0.134 |
| No | 20 (90.9) | 1,131 (77.5) |  |
| Yes | 2 (9.1) | 328 (22.5) |  |
| *Missing* | *0* | *1* |  |
| **Month of study visit 3 (in 2022)** |  |  | 0.012 |
| May | 0 (0.0) | 40 (2.7) |  |
| June | 11 (50.0) | 303 (20.8) |  |
| July | 4 (18.2) | 476 (32.6) |  |
| August | 2 (9.1) | 302 (20.7) |  |
| September | 3 (13.6) | 225 (15.4) |  |
| October | 0 (0.0) | 73 (5.0) |  |
| November | 2 (9.1) | 41 (2.8) |  |

Abbreviations: *HELIUS* Healthy Life in an Urban Setting; *SARS-CoV-2* Severe acute respiratory syndrome coronavirus 2; *IQR* interquartile range. ^a^ Measured at HELIUS baseline (2011–2015). ^b^ Age was recalculated for the third COVID-19 substudy visit. ^c^ Higher education level includes higher vocational schooling and university. ^d^ Participants were classified as being more integrated into the host society when not applicable (Dutch ethnic origin) or when measured to be integrated or assimilated; participants were classified as less integrated when measured to be separated or marginalized, according to Berry's acculturation strategies (reference: Berry JW. Immigration, Acculturation, and Adaptation. Applied Psychol: An International Review 1997;46:5–68). ^e^ Measured during the third COVID-19 substudy visit (May-November 2022). ^f^ SARS-CoV-2 vaccination status was determined by the question “Which primary vaccinations have you received?”. Incomplete: received one dose of a vaccine other than Janssen, with or without subsequent infection; complete: received two doses of Pfizer, Moderna or AstraZeneca, ≥1 dose of Janssen, or had a past infection and subsequently received ≥1 dose of any vaccine (based on the guidelines of the Dutch government, reference: National Institute for Public Health and the Environment. COVID-19-vaccinatie uitvoeringsrichtlijn - version 4 December 2021. 2021. Available from: <https://lci.rivm.nl/richtlijnen/covid-19-vaccinatie>. Accessed on: 20 March 2023). ^g^ Booster status was determined by the question “Have you received a booster vaccination?”. ^h^ Measured using WANTAI ELISA during the first (June-October 2020) and second (November 2020-June 2021) COVID-19 substudy visit.


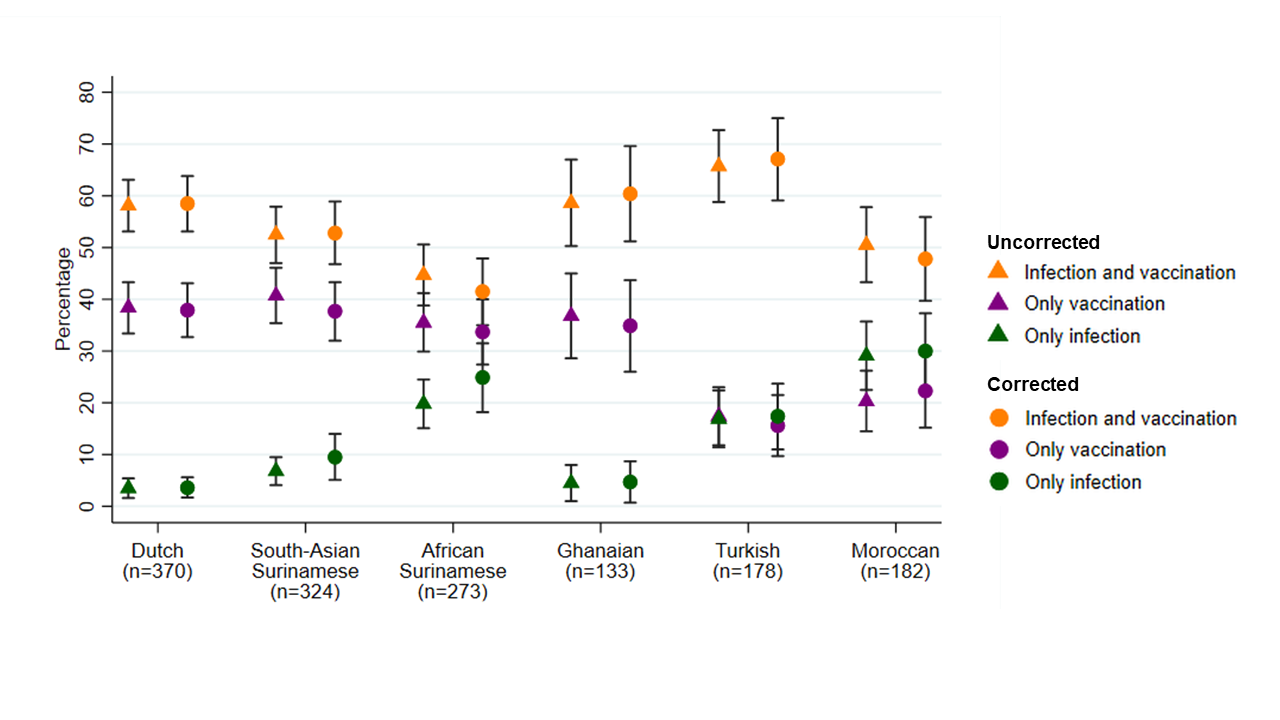


**Supplementary Figure S3.** Uncorrected and corrected SARS-CoV-2 infection and vaccination status ^a^ among HELIUS participants with a positive SARS-CoV-2 WANTAI ELISA antibody test result, per ethnic group, Amsterdam, the Netherlands, May 17, 2022 - November 21, 2022 (n=1,460). Abbreviations: *SARS-CoV-2* Severe acute respiratory syndrome coronavirus 2; *HELIUS* Healthy Life in an Urban Setting; *ELISA* enzyme-linked immunosorbent assay. ^a^ Prior infection and vaccination status was defined as being only previously vaccinated (based on the self-reported uptake of ≥1 SARS-CoV-2 vaccine dose, without evidence of prior SARS-CoV-2 infection), only previously infected (based on having a positive antibody test result at the third COVID-19 substudy visit without reporting to be previously vaccinated), or both previously infected and vaccinated [based on a positive SARS-CoV-2 antibody test result during the second substudy visit (November 2020-June 2021), or the first substudy visit (June-October 2020) if the test result from the second substudy visit was unavailable, or on self-report at the third substudy visit when previous antibody test results were negative or missing]. The triangles and circles represent the uncorrected and corrected estimated infection and vaccination status, respectively, and bands the corresponding 95% confidence interval. Corrected infection and vaccination status estimates account for the age and sex distribution of the Amsterdam population through post-stratification weights and for sampling.

**Supplementary Table S3.** Association between ethnicity and SARS-CoV-2 vaccination and infection status ^a^, while accounting for sociodemographic, access to healthcare, and acculturation determinants, among HELIUS participants with a positive SARS-CoV-2 WANTAI ELISA antibody test result, Amsterdam, the Netherlands, May 17, 2022 - November 21, 2022 ^b^.

|  | **Univariable model** | |  | **Multivariable model 1 (n=1,425)** | |  | **Multivariable model 2 (n=1,033)** | |  |
| --- | --- | --- | --- | --- | --- | --- | --- | --- | --- |
|  | **Only vaccinated vs. infected and vaccinated** | **Only infected vs. infected and vaccinated** |  | **Only vaccinated vs. infected and vaccinated** | **Only infected vs. infected and vaccinated** |  | **Only vaccinated vs. infected and vaccinated** | **Only infected vs. infected and vaccinated** |  |
|  | OR (95% CI) | OR (95% CI) | *P* value | aOR (95% CI) | aOR (95% CI) | *P* value | aOR (95% CI) | aOR (95% CI) | *P* value |
| **Ethnicity** |  |  | <0.001 |  |  | <0.001 |  |  |  |
| Dutch | Ref | Ref |  | Ref | Ref |  | **-** | **-** |  |
| South-Asian Surinamese | 1.10 (0.79-1.53) | 2.91 (1.34-6.33) |  | 1.28 (0.89-1.85) | 3.31 (1.50-7.31) |  | Ref | Ref | <0.001 |
| African Surinamese | 1.25 (0.86-1.81) | 9.68 (4.89-19.15) |  | 1.27 (0.84-1.93) | 10.41 (5.17-20.94) |  | 0.96 (0.62-1.47) | 3.28 (1.67-6.43) |  |
| Ghanaian | 0.89 (0.56-1.40) | 1.25 (0.43-3.65) |  | 1.15 (0.65-2.05) | 2.68 (0.75-9.58) |  | 0.86 (0.47-1.60) | 0.79 (0.23-2.67) |  |
| Turkish | 0.36 (0.21-0.60) | 4.18 (2.01-8.67) |  | 0.55 (0.30-1.00) | 3.74 (1.52-9.20) |  | 0.40 (0.22-0.75) | 1.27 (0.57-2.83) |  |
| Moroccan | 0.72 (0.44-1.17) | 10.11 (5.12-19.96) |  | 1.06 (0.57-1.96) | 15.24 (6.70-34.65) |  | 0.82 (0.44-1.52) | 3.57 (1.73-7.35) |  |
| **Age in years** |  |  |  |  |  | 0.004 |  |  | <0.001 |
| <45 | Ref | Ref |  | Ref | Ref |  | Ref | Ref |  |
| 45-54 | 0.74 (0.40-1.37) | 1.05 (0.60-1.83) |  | 0.77 (0.41-1.44) | 0.87 (0.44-1.71) |  | 0.82 (0.42-1.59) | 0.67 (0.36-1.24) |  |
| 55-59 | 1.45 (0.80-2.64) | 0.35 (0.17-0.71) |  | 1.44 (0.79-2.62) | 0.41 (0.19-0.88) |  | 1.41 (0.72-2.74) | 0.36 (0.17-0.73) |  |
| ≥60 | 1.93 (1.16-3.20) | 0.58 (0.32-1.05) |  | 1.77 (1.06-2.96) | 0.86 (0.46-1.63) |  | 2.06 (1.11-3.81) | 0.36 (0.17-0.73) |  |
| **Female vs. male sex** | 0.71 (0.51-1.00) | 1.41 (0.91-2.19) |  | 0.73 (0.51-1.05) | 1.25 (0.75-2.09) | 0.106 | 0.85 (0.55-1.32) | 1.01 (0.61-1.67) | 0.752 |
| **Household size** |  |  |  |  |  | 0.370 |  |  |  |
| 1 | Ref | Ref |  | Ref | Ref |  | Ref | Ref |  |
| 2 | 0.66 (0.42-1.04) | 0.87 (0.44-1.69) |  | 0.59 (0.37-0.96) | 0.95 (0.44-2.07) |  | 0.98 (0.54-1.77) | 0.67 (0.30-1.52) |  |
| 3 | 0.68 (0.39-1.21) | 0.94 (0.47-1.89) |  | 0.76 (0.42-1.39) | 0.71 (0.33-1.53) |  | 0.80 (0.44-1.45) | 0.80 (0.36-1.82) |  |
| 4 | 0.50 (0.28-0.89) | 0.74 (0.35-1.53) |  | 0.60 (0.32-1.10) | 0.59 (0.25-1.38) |  | 0.51 (0.28-0.91) | 0.54 (0.25-1.18) |  |
| ≥5 | 0.36 (0.20-0.66) | 1.66 (0.85-3.25) |  | 0.51 (0.26-1.00) | 0.65 (0.28-1.50) |  | 0.68 (0.35-1.34) | 0.66 (0.30-1.46) |  |
| **Level of trust in the government pandemic response** |  |  |  |  |  | <0.001 |  |  | <0.001 |
| Trust | Ref | Ref |  | Ref | Ref |  | Ref | Ref |  |
| Neutral | 0.81 (0.56-1.18) | 3.98 (2.23-7.09) |  | 0.90 (0.61-1.33) | 2.97 (1.64-5.37) |  | 0.91 (0.58-1.43) | 2.02 (1.05-3.90) |  |
| No trust | 1.00 (0.57-1.78) | 14.09 (7.19-27.62) |  | 1.09 (0.59-2.02) | 13.67 (6.37-29.32) |  | 0.78 (0.35-1.74) | 5.00 (2.33-10.76) |  |
| **Cultural orientation, less integrated ^c^** | 0.99 (0.57-1.73) | 1.16 (0.61-2.22) | 0.886 | - | - | - | 1.26 (0.69-2.31) | 1.43 (0.70-2.92) | 0.512 |
| **Low health literacy** | 0.54 (0.29-1.01) | 0.24 (0.10-0.59) | 0.003 | - | - | - | 0.60 (0.28-1.26) | 0.39 (0.13-1.15) | 0.127 |
| **Month of study visit 3 (May - Nov 2022) ^d^** | 0.81 (0.72-0.92) | 1.08 (0.93-1.25) |  | 0.87 (0.75-0.99) | 0.93 (0.77-1.12) | 0.120 | 1.05 (0.88-1.27) | 1.00 (0.82-1.22) | 0.853 |

Abbreviations: *SARS-CoV-2* Severe acute respiratory syndrome coronavirus 2; *HELIUS* Healthy Life in an Urban Setting; *ELISA* enzyme-linked immunosorbent assay; *OR* Odds ratio; *aOR* Adjusted odds ratio; *CI* Confidence interval; *Ref* Reference category. ^a^ Prior infection and vaccination status was defined as being only previously vaccinated (based on the self-reported uptake of ≥1 SARS-CoV-2 vaccine dose, without evidence of prior SARS-CoV-2 infection), only previously infected (based on having a positive antibody test result at the third COVID-19 substudy visit without reporting to be previously vaccinated), or both previously infected and vaccinated (based on the self-reported uptake of at least one SARS-CoV-2 vaccine dose and having tested seropositive during previous substudy visits [visit 1: June-October 2020 or visit 2: November 2020-June 2021] or, if antibody test results during previous visit were negative or unavailable, on self-reported prior infection). ^b^ Analyses were performed using multinomial logistic regression (reference=both infected and vaccinated). Observations with missing values on covariates were removed from analysis. Analyses account sampling, and for the age and sex distribution of the Amsterdam population through post-stratification weights. Model 1: Adjusted for age, sex, household size, level of trust in the government’s response to the pandemic, month of study visit 3. Model 2: Adjusted for age, sex, household size, level of trust in the government´s response to the pandemic, cultural orientation, health literacy, month of study visit 3, while excluding those with a Dutch ethnic background. ^c^  Separation, marginalization. ^d^ Month of study visit was modelled as a continuous variable, with consecutive integers beginning at 1 for May 2022 and ending at 7 for November 2022.

**Supplementary Table S4**. Association between ethnicity and SARS-CoV-2 vaccination and infection status ^a^ among HELIUS participants with a positive SARS-CoV-2 WANTAI ELISA antibody test result who had antibody test results from all three COVID-19 substudy visits, Amsterdam, the Netherlands, May 17, 2022 - November 21, 2022 (sensitivity analysis) ^b^.

|  | **Univariable model** | |  | **Multivariable model 1 (n=1,337)** | |  | **Multivariable model 2 (n=966)** | |  |
| --- | --- | --- | --- | --- | --- | --- | --- | --- | --- |
|  | **Only vaccinated vs. infected and vaccinated** | **Only infected**  **vs. infected and vaccinated** |  | **Only vaccinated vs. infected and vaccinated** | **Only infected**  **vs. infected and vaccinated** |  | **Only vaccinated vs. infected and vaccinated** | **Only infected vs. infected and vaccinated** |  |
|  | OR (95% CI) | OR (95% CI) | *P* value | aOR (95% CI) | aOR (95% CI) | *P* value | aOR (95% CI) | aOR (95% CI) | *P* value |
| **Ethnicity** |  |  | <0.001 |  |  | <0.001 |  |  | <0.001 |
| Dutch | Ref | Ref |  | Ref | Ref |  | **-** | **-** |  |
| South-Asian Surinamese | 1.09 (0.77-1.53) | 3.91 (1.69-9.00) |  | 1.33 (0.91-1.94) | 4.52 (1.98-10.35) |  | Ref | Ref |  |
| African Surinamese | 1.20 (0.82-1.75) | 10.90 (5.12-23.22) |  | 1.25 (0.81-1.92) | 12.22 (5.88-25.39) |  | 0.96 (0.62-1.40) | 2.79 (1.41-5.53) |  |
| Ghanaian | 0.91 (0.56-1.46) | 1.20 (0.33-4.35) |  | 1.21 (0.66-2.21) | 2.65 (0.63-11.23) |  | 0.89 (0.47-1.69) | 0.53 (0.13-2.09) |  |
| Turkish | 0.34 (0.20-0.57) | 5.16 (2.32-11.47) |  | 0.54 (0.28-1.01) | 5.12 (2.03-12.93) |  | 0.38 (0.20-0.71) | 1.11 (0.49-2.51) |  |
| Moroccan | 0.66 (0.40-1.10) | 11.23 (5.30-23.77) |  | 0.99 (0.52-1.91) | 18.91 (8.07-44.28) |  | 0.72 (0.37-1.40) | 3.08 (1.47-6.45) |  |

Abbreviations: *SARS-CoV-2* Severe acute respiratory syndrome coronavirus 2; *HELIUS* Healthy Life in an Urban Setting; *ELISA* enzyme-linked immunosorbent assay; *COVID-19* Coronavirus disease 2019; *OR* Odds ratio; *aOR* Adjusted odds ratio; *CI* Confidence interval; *Ref* Reference category. ^a^ Infection and vaccination status was defined as being only previously vaccinated (based on the self-reported uptake of ≥1 SARS-CoV-2 vaccine dose, without evidence of prior SARS-CoV-2 infection), only previously infected (based on having a positive antibody test result at the third COVID-19 substudy visit without reporting to be previously vaccinated), or both previously infected and vaccinated (based on the self-reported uptake of at least one SARS-CoV-2 vaccine dose and having tested seropositive during previous substudy visits [visit 1: June-October 2020 or visit 2: November 2020-June 2021] or, if antibody test results during previous visit were negative or unavailable, on self-reported prior infection). ^b^ Analyses were performed using multinomial logistic regression (reference=both infected and vaccinated). Analyses account sampling, and for the age and sex distribution of the Amsterdam population through post-stratification weights. Model 1: Adjusted for age, sex, household size, level of trust in the government´s response to the pandemic, month of study visit 3. Model 2: Adjusted for age, sex, household size, level of trust in the government´s response to the pandemic, cultural orientation, health literacy, month of study visit 3, while excluding those with a Dutch ethnic background.

**Supplementary Table S5.** E-value analysis assessing the minimum strength of association of unmeasured confounders needed to explain the observed association between ethnicity and SARS-CoV-2 infection and vaccination status.

Model 1 (adjusted for age, sex, household size, and month of study visit)

|  | **Only vaccinated vs. infected and vaccinated** | **Only infected vs. infected and vaccinated** |
| --- | --- | --- |
|  | **E-value** | **E-value** |
| **Ethnicity** |  |  |
| Dutch | Reference group | Reference group |
| South-Asian Surinamese | 1.88 | 6.08 * |
| African Surinamese | 1.86 | 20.31 * |
| Ghanaian | 1.57 | 4.80 |
| Turkish | 3.04 | 6.94 * |
| Moroccan | 1.31 | 29.97 * |

* The association between the ethnic group and being only infected versus infected and vaccinated, adjusted for age, sex, household size, and month of study visit, was significantly different from the Dutch origin group (*P*<0.05).

Model 2 (adjusted for age, sex, household size, cultural orientation, health literacy, and month of study visit; excluding individuals of Dutch origin)

|  | **Only vaccinated vs. infected and vaccinated** | **Only infected vs. infected and vaccinated** |
| --- | --- | --- |
|  | **E-value** | **E-value** |
| **Ethnicity** |  |  |
| South-Asian Surinamese | Reference group | Reference group |
| African Surinamese | 1.25 | 6.01 * |
| Ghanaian | 1.60 | 1.85 |
| Turkish | 4.44 * | 1.86 |
| Moroccan | 1.74 | 6.60 * |

* The association between the ethnic group and being only infected versus infected and vaccinated, adjusted for age, sex, household size, cultural orientation, health literacy, and month of study visit, was significantly different from the South-Asian Surinamese origin group (*P*<0.05).
